# Supplementary material for: Prescribed hormonal contraceptive use trends in the Estonian Biobank: A longitudinal observational study
Source: PLoS Med. 2026 May 20;23(5):e1005086. doi: 10.1371/journal.pmed.1005086 (PMC13221152; doi:10.1371/journal.pmed.1005086)
Supplement: Attachment — Submitted filename: dzigurski.pdf [file pmed.1005086.s009.pdf]

This paper uses various existing databases to analyse the usage of hormonal contraception by women in Estonia.

I was asked for a methodological report and I interpret that to include all aspects of the design and conduct of the study.

Points of detail

Page 3 onwards Not really a statistical point but are so many references needed in the introduction? There are often three or four referring to the same detail.

Page 6 What percentage of the Estonian population is covered by the insurance fund?

Page 7 Did the age restriction only apply per purchase/prescription so that a woman could be in the cohort for other occasions when she was age–eligible?

Page 7 13.5, 19.5, 52 seems an odd progression especially when it is going to be reduced to 3, 5 and 6. Is there a typo here?

Page 7 I am afraid I do not understand the sentence starting 'HC usage period'. What does it mean for them to be adjusted? What is the force of the phrase 'within the original coverage period'?

Page 7 I do not understand the sentence starting 'Furthermore, for HC'. This may reflect my general ignorance of hormonal contraception.

Page 8 If I understand additional figure 1 correctly this would provide valuable information about how people are classified. However it tries to put much information onto one page with the result that it needs blowing up to read clearly. In turn that means you have to scroll repeatedly to get the information. I suggest that the section which has some similarity to a Gantt chart (towards the top) be made a separate figure and moved into the main text. The caption could also give more detail.

Page 11 It is clearly a good idea to credit the software used but just stating R base and the tidyverse is not that helpful. Base R contains many functions if we include the stats and graphics packages and the main tidyverse package downloads many functions which do the actual work. I would just cite the key packages used for analysis with proper citations which, as I am sure the authors are aware, can be found on CRAN or by using the citation() function.

Page 11 It might be helpful to give some quantiles for variables like age. One thing which seems to be missing from the write–up is a table of demographic characteristics. Incidentally I do not think presenting tables in spreadsheets is appropriate for publication compared to properly typeset tables. By all means give us data in machine–readable format as well.

Page 16 What definition of outlier is being used here? If these are boxplot outliers then is that sensible with distributions which do not seem symmetrical (on the log scale)? The caption does not mention what I take to be the region between the upper and lower quartile.

Page 21 We really need estimates of differences with confidence intervals for all of these. The same applies on page 2. This applies especially to those which failed to reach a conventional level of statistical significance (Chan, 2013). Editors of health journals started calling for measures of effect size instead of p–values more than three decades ago (Langman, 1986; Gardner and Altman, 1986) For some reason sensitivity and specificity do, correctly, have confidence intervals.

Page 21 Why use Fisher's exact test for such large numbers?

Page 27 I do not understand the apparent conflict between different medications for emergency prescription, over the counter versus on prescription only.

The appropriate reporting guideline here would seem to be RECORD (Benchimol et al., 2015). I have not checked for compliance with all the individual items in the checklist.

Point of more substance

This is a longitudinal dataset but there are features of the design which do not seem to have been taken into account. The percentages quoted throughout do not seem to have been adjusted for the fact that a woman may appear multiple times and so those observations are not independent. Also women may appear in the dataset in different age categories as they give observations and that needs attention too. If, in fact, the analysis did take that into account then the methods section needs to specify how it was done.

Summary

Several points for clarification and a more important one about the relationship between the design and the analysis.

Michael Dewey

References

E I Benchimol, L Smeeth, A Guttmann, K Harron, D Moher, I Petersen, H Sørensen, E von Elm, S Langan, and RECORD working committee. The REporting of studies Conducted using Observational Routinely–collected health Data(RECORD) Statement. *PLOS Medicine*, 12(10), 2015.

L S Chan. Minimal clinically important difference MCID adding meaning to statistical inference. *American Journal of Public Health*, 103:e24–e25, 2013.

M J Gardner and D G Altman. Confidence intervals rather than P values: estimation rather than hypothesis testing. *British Medical Journal*, 292: 746–750, 1986.

M J S Langman. Towards estimation and confidence intervals. *British Medical Journal*, 292:716, 1986.
