# Supplementary material for: Prescribed hormonal contraceptive use trends in the Estonian Biobank: A longitudinal observational study
Source: PLoS Med. 2026 May 20;23(5):e1005086. doi: 10.1371/journal.pmed.1005086 (PMC13221152; doi:10.1371/journal.pmed.1005086)
Supplement: Attachment — Submitted filename: Response_to_Reviewers_auresp_3.docx [file pmed.1005086.s012.docx]

Prescribed hormonal contraceptive use trends in the Estonian Biobank: A longitudinal observational study

Jelisaveta Džigurski^1^, Märt Möls^1,2^, Kristi Läll^1^, Hannah Currant^3,4^, Mall Eltermaa^5^, Estonian Biobank Research Team^1^, Reedik Mägi^1^, Lili Milani^1^, Triin Laisk^1,*^

^*^Corresponding author

^1^Estonian Genome Centre, Institute of Genomics, University of Tartu, Riia Street 23b, Tartu, 51010, Estonia

^2^Institute of Mathematics and Statistics, University of Tartu, Narva 18, Tartu, 51009, Estonia

^3^Nuffield Department of Women’s and Reproductive Health, University of Oxford, Old Road Campus, Oxford, OX3 7LF, UK

^4^Big Data Institute, University of Oxford, Old Road Campus, Oxford, OX3 7LF, UK

^5^Institute of Biomedicine and Translational Medicine, University of Tartu, Ravila Street 19, Tartu, 50411, Estonia

# Revision 2

**We thank the Academic Editor, Editors and Editorial Team, and Reviewers for their diligent review and detailed reading of the manuscript. Your suggestions and comments have substantially improved the manuscript, making our work clearer for the readership.**

# Academic Editor

Line 530:"We note that the prevalence rates...." Is prevalence the correct term? Ed: consider usage rates? Please check use of prevalence throughout for accuracy of the term.

We thank the Academic Editor for raising this point and we appreciate the suggestion of ‘usage rate’ as an alternative. After careful consideration, we decided to keep ‘annual prevalence’ (without the ‘rate’), as this term is well established in the epidemiological literature and accurately reflects the calculation we used (annual prevalence proportion as the number of individuals who used the drug in a year divided by the study population size in the same year). We hope this change throughout the manuscript helps resolve the issue and is acceptable to the Academic Editor.

# Editors

Please check the grammar throughout, including the Abstract, and revise, correcting errors (e.g. lines 17, 19, 29, 34).

Thank you for this comment. We have now carefully revised the entire manuscript for grammar and language and corrected the errors.

Please ensure that the statements in the metadata are up to date and match the statements in the manuscript files.

We have now corrected discrepancies between the metadata and the manuscript file statements, and confirm that all fields are now consistent.

Please amend the COI for HC as the funding does not constitute a COI. Instead, please amend the funder statement for HC who declares funding from the Wellcome and the NIH.

We have now removed COI for HC and added to the funding statement: “HC is funded by the Wellcome Trust (https://wellcome.org/) 318918/Z/24/Z **and the National Institutes of Health (NIH: https://www.nih.gov) 1P50HD104224-01**.”.

Please amend the COI statement for KL to match the declaration in the questionnaire.

We have now added: “**We have read the journal’s policy and the authors of this manuscript have the following competing interests: KL has participated as an analyst in a research grant awarded to the Institute of Genomics from Geneto OÜ. All other authors have declared that they have no competing interests.**”.

Please add URLs for each funder to the funding statement. The funding statement should include: specific grant numbers, initials of authors who received each award, URLs to sponsors’ websites. Also, please state whether any sponsors or funders (other than the named authors) played any role in study design, data collection and analysis, the decision to publish, or preparation of the manuscript. If they had no role in the research, include this sentence: “The funders had no role in study design, data collection and analysis, decision to publish, or preparation of the manuscript.”

We have now updated the funding statement according to your requests as follows: “**TL and JD are funded by the Estonian Research Council (https://etag.ee/en/) grants TK214 and PSG776. MM is funded by the Estonian Research Council (https://etag.ee/en/) grant PRG1911. RM and KL are funded by the Estonian Research Council (https://etag.ee/en/) grants TK214 and PRG1911. LM is funded by the Estonian Research Council (https://etag.ee/en/) grant PRG2625. HC is funded by the Wellcome Trust (https://wellcome.org/) 318918/Z/24/Z and the National Institutes of Health (NIH: https://www.nih.gov) 1P50HD104224-01. The funders had no role in study design, data collection and analysis, decision to publish, or preparation of the manuscript.**”.

We require deposition of code in a public repository and that a DOI is generated. Links to the code must be provided in the final version of the manuscript (see below).

We have now uploaded the code to GitHub and permanently archived it on Zenodo with a citable DOI. The statement is as follows: “**The source code used in this study is publicly available from GitHub (https://github.com/alterecho11/hc_use_estbb) and archived on Zenodo (https://doi.org/10.5281/zenodo.19615879).**”.

Line 666: is consent 'informed consent'? If so, please add the qualifier.

Yes, we have now clarified: “All participants have signed a broad **informed** consent form.”. In addition, we have moved the “Ethics approval and consent to participate” section from the Declarations to the Methods section.

Members of the Estonian Biobank Research Team should be identified. As they are authors, they should not be included in the Acknowledgments section but identified separately and their author contributions stated in the Author Contribution statement. Please note that author RM has not completed the questionnaire with the COI declaration.

The Estonian Biobank Research Team is a banner/consortium author. Two of its members, RM and LM, are listed as individual authors due to their direct involvement in this work. The remaining members contributed collectively and indirectly. According to your last e-mail, the list of consortium authors should be included in the Acknowledgements. We have now added the remaining consortium authors to the Acknowledgements section: “The authors thank the Estonian Biobank participants for their contribution to this work, and the **Estonian Biobank Research Team members** (**Andres Metspalu, Tõnu Esko, Mait Metspalu, Mari Nelis and Georgi Hudjashov**) for data collection, genotyping, QC and imputation.”. We confirm that RM has completed the author questionnaire.

All authors must declare their relevant competing interests per the PLOS policy, which can be seen here: <https://journals.plos.org/plosmedicine/s/competing-interests> For authors with ties to industry, please indicate whether any of the interests has a financial stake in the results of the current study.

The Estonian Biobank Research Team is a banner/consortium author. Two of its members, RM and LM, are listed as individual authors due to their direct involvement in this work. Accordingly, they have completed the author questionnaire and declared their COI. The remaining members contributed collectively and indirectly and have no COI or funding relevant to this study. As for the previous point, we hope this clarification is acceptable, and we are happy to provide any further information if needed.

In the final bullet point of the Author Summary section ‘What Do These Findings Mean?’ Please include the main limitations of the study in non-technical language.

Thank you for bringing this to our attention. We have now revised the whole Author Summary section ‘What Do These Findings Mean?’ as follows:

- This study highlights the potential of the EstBB dataset for HC research and offers a reproducible framework.
- Population-based biobanks with genetic and health-related data, such as the EstBB, offer a valuable resource for studies investigating pharmacogenetic aspects of HCs.
- **This study relies on records of HC purchases accumulated over a long period, and from these records alone, we could not confirm whether HCs were used as purchased. Diagnoses identified near the HC usage period may not represent true side effects. Also, because HC use varies across countries, our findings may not apply to women residing outside Estonia.**

Please confirm that your title complies with PLOS Medicine's style. Your title must be nondeclarative and not a question. It should begin with main concept if possible. "Effect of" should be used only if causality can be inferred, i.e., for an RCT. Please place the study design ("A randomized controlled trial," "A retrospective study," "A modelling study," etc.) in the subtitle (ie, after a colon). We suggest: "Prescribed hormonal contraceptive use trends and genetic associations with side effects in the Estonian Biobank: A longitudinal observational study".  Please amend accordingly, retaining the style.

Thank you for this helpful suggestion. We have now changed the title to: “**Prescribed hormonal contraceptive use trends in the Estonian Biobank: A longitudinal observational study**”.

Please confirm that your abstract complies with our requirements, including format (three sections: Background, Methods and Findings, and Conclusions) and providing all the information relevant to this study type <https://journals.plos.org/plosmedicine/s/submission-guidelines#loc-abstract>

We confirm that we have included all three relevant sections (Background, Methods and Findings, and Conclusions) in the newly revised version. We now clearly present the background and study rationale, outline the methods and key findings, and provide a concise interpretation of the results, limitations and implications for future research.

Please ensure that the Introduction ends with a clear description of the study question or hypothesis.

Thank you for bringing this to our attention. We have now stated concisely our study question: “**Overall, we aim to investigate whether the EstBB dataset is suitable for future genetic studies of HC-related side effects and disease risk.**”.

Please ensure that all abbreviations are defined at first use throughout the text.

We confirm that all abbreviations are defined at their first use throughout the Abstract, Author Summary and main text.

Please confirm that all numbers presented in the abstract are present and identical to numbers presented in the main manuscript text.

We confirm that the numbers in the Abstract and the main text are identical.

Abbreviations in figures need to be spelled out in the legends. VTE/ATE in figure 5, COC in figure 2.

Thank you for bringing this to our attention. We have revised the manuscript, and all abbreviations in figures, tables, and supplementary information are now defined in the titles and legends.

Please add a title for the y axis of Fig. 3, identifying the abbreviations as HC formulations.

We have now added the title for the y-axis of Fig. 3, defined abbreviations and called out Box 1 (see below) to help with HC formulation abbreviations as follows: “Length of first observed hormonal contraceptive (HC) usage period for different HC formulations. **Specific HC formulations are listed on the y-axis (see Box 1 for definitions),** while the HC formulation usage period length in days (days shown on the logarithmic scale) is plotted along the x-axis. The colour illustrates the HC type. The box represents the interquartile range (25th to 75th percentile), with the central diamond shapes marking the median, and the black dots marking outliers. **Abbreviations: IUD – intrauterine device; COC – combined oral contraceptive; CHC – combined hormonal contraceptive; (s) – CHC pill with sequential preparations; (p) – transdermal patch**.”

Please add a Box with the list of HC formulation abbreviations and their definitions and add a call out to the Box in the text.

Thank you for this helpful suggestion. We have now added: “**Box 1. Hormonal contraceptive abbreviations used in the study.**”.

If statistical tests are used to generate data in figures, please identify the tests in the figure legends.

No statistical tests were used to generate data in the figures. The dummy data provided was created manually to mirror the structure of the real input, with no statistical properties derived from the real data. We have now clarified: “**The manually defined data points are for illustrative purposes and are not real observations**.”.

In general we prefer that authors convert bar graphs to another format. If this is not feasible or impairs presentation, please include in the SI Tables with the data behind the bar graphs for figures 2c and 4c. Please also note that it is difficult to discern between the blue and purple bars. Please alter the intensities for better distinction.

Thank you for the opportunity to clarify this point. We evaluated alternative visualisations for stacked bar graphs, such as heatmaps and dot plots, and concluded that these alternatives introduce greater confusion without improving interpretability. While stacked bar graphs are not ideal for detailed comparisons, they are useful for illustrating the overall trends across many years and many HC types. To facilitate precise comparisons, we reported all underlying data in SI Tables. Specifically, Fig 2C is supported by Table D in S3 Appendix, and Fig 4C is supported by Table F in S3 Appendix. We have changed the colour intensities in the palette to improve clarity and distinction between hormonal contraceptive (HC) types.

Please review your text for claims of novelty or primacy (e.g. 'for the first time') and remove this language. In addition, please check that any use of statistical terms (such as trend or significant) are supported by the data, and if not please remove them.

We have now removed all novelty claims from the Discussion. However, we wish to clarify that the term “first-time” is retained in specific instances where it serves a descriptive purpose to distinguish the *first-time observed HC usage period* of an individual in our study cohort. This distinction is necessary to avoid misinterpretation from the first use of a specific HC formulation, or the first use of a specific HC formulation in a given year. The same applies to first-time records of diagnosis codes. We hope you agree that this usage is appropriate in context. Where the data could support statistical terms, we have added references to the relevant figures and tables. Where they could not, we have removed them.

In the last sentence of the Abstract Methods and Findings section, please describe the main limitation(s) of the study's methodology.

We have now clarified in the abstract: “**The study was limited by the use of administrative medication purchase records, which may not reflect actual HC use. Additionally, diagnoses identified near the HC usage period may not represent true side effects.**”.

In the abstract, please include the important dependent variables that are adjusted for in the analyses.

We note that no dependent variables were adjusted for, as all analyses in the study were descriptive. The term “adjusted” was previously used in a non-statistical context, which may have led to misunderstanding. To prevent confusion, we have removed this terminology and hope this revision addresses your concern.

In the author summary, in the final bullet point of 'What Do These Findings Mean?', please include the main limitations of the study in non-technical language.

We have now added to the Author Summary section ‘What Do These Findings Mean?’:

- **This study relies on records of HC purchases accumulated over a long period, and from these records, we could not confirm whether HCs were used as purchased. Diagnoses identified near the HC usage period may not represent true side effects. Also, because HC use varies across countries, our findings may not apply to women residing outside Estonia.**

Authors are responsible for providing the source code needed to replicate the study's findings in a repository (such as GitHub, SourceForge or Bitbucket) or a cloud computing service (such as Code Ocean). Protection of authors’ intellectual property will not be cause for exception. Please explain in the manuscript’s Data Availability Statement how readers can access the shared code.

We have now uploaded the source code to GitHub and permanently archived it on Zenodo with a citable DOI. We explained in the Data Availability statement: “**The source code used in this study is publicly available from GitHub (https://github.com/alterecho11/hc_use_estbb) and archived on Zenodo (https://doi.org/10.5281/zenodo.19615879).**”.

Please include the statement on code availability in the data availability statement.

Yes, we have now included the statement on code availability in the Data Availability statement: “**The source code used in this study is publicly available from GitHub (https://github.com/alterecho11/hc_use_estbb) and archived on Zenodo (https://doi.org/10.5281/zenodo.19615879).**”.

Please provide titles and legends for all figures and tables (including those in Supporting Information files). Please define all acronyms used in each figure or table in its corresponding legend.

We confirm that all figures and tables, including those in the Supporting Information, now include titles and legends. In addition, all acronyms are defined within their legends.

Please convert any stacked bar charts to another data representation for example a table, or other type of graph (see above).

We appreciate the suggestion. We would like to keep the stacked bar charts in Figures 2C and 4C, as these are suitable for illustrating overall trends across the many study years and HC types. We found alternative visualisations to be less interpretable. To support precise comparisons, we have reported all underlying data in the Supporting Information: Figure 2C is supported by Table D in S3 Appendix, and Figure 4C is supported by Table F in S3 Appendix. We hope that you will approve the use of stacked bar graphs, given that the underlying data is transparently provided.

Please provide the unadjusted comparisons as well as the adjusted comparisons in all relevant Tables

All analyses in this study are descriptive, and no dependent variables were adjusted for. The term “adjusted” was previously used in a non-statistical context, which may have confused readers. We have now removed this terminology throughout the manuscript to prevent further misunderstanding. In the Table H in S3 Appendix where we account for multiple testing, we report both the original (uncorrected) and multiple-testing-corrected p-values.

Please update the STROBE and RECORD checklists is necessary. When completing the checklists, please use section and paragraph numbers, rather than page numbers.

We confirm that both STROBE and RECORD checklist items correspond to the relevant paragraph in the manuscript where the information is reported.

Your study is observational and therefore causality cannot be inferred. Please remove language that implies causality and refer to associations instead.

Thank you for raising this important point. We have revised the manuscript and removed all language that could imply a causal relationship.

For all observational studies, in the manuscript text, please indicate: (1) the specific hypotheses you intended to test, (2) the analytical methods by which you planned to test them, (3) the analyses you actually performed, and (4) when reported analyses differ from those that were planned, transparent explanations for differences that affect the reliability of the study's results. If a reported analysis was performed based on an interesting but unanticipated pattern in the data, please be clear that the analysis was data-driven.

Thank you for this suggestion. We have clarified in the Methods section: “**This is a descriptive study without a predefined hypothesis,** aiming to comprehensively characterise observed HC use in the EstBB and evaluate its potential for future genetic analyses of HC-related side effects and disease risk. The study is reported according to the Strengthening the Reporting of Observational Studies in Epidemiology (STROBE) guideline (Table A in S1 Checklist) and the Reporting of Studies Conducted using Observational Routinely Collected Data (RECORD) guideline (Table B in S1 Checklist) [32,33]. **The study did not have a prospective protocol or analysis plan. In response to peer review suggestions, one additional analysis was completed: evaluation of thromboembolism risk factors among POC users and CHC users stratified by estrogen dose [34].**”.

# Reviewer #1

Thank you for submitting your revision. In reviewing the comments to the reviewers and the changes that the authors made to this publication as a result, I am happy to provide support to accept. I am happy to see that scientists are thinking about longtiduinal biobanks with contraceptive realted data. This will undoubetly support research in this area.

Thank you for your time, insightful comments and careful evaluation of our manuscript. We are happy to read that you have positively assessed our work and acknowledged the importance of the research topic.

# Reviewer #2

Thank you very much for the opportunity to review the revised manuscript. The revisions have addressed the outstanding issues, and the current version is now more clearly structured; the remaining questions have been adequately addressed. No further changes are required.

Thank you for your time and for your thorough, thoughtful review. We are pleased to read that the current version is well-structured and that we adequately addressed your questions.

# Reviewer #3

The authors have addressed all my comments.

Thank you for reviewing our manuscript and for your helpful comments. We are pleased to read that we successfully addressed all of your comments.

# Reviewer #4

The authors have addressed my points.

Michael Dewey

Thank you for your time and valuable constructive feedback throughout the review process. We are pleased to read that we successfully addressed your points.

# Technical Requests

1) Thank you for stating "The code used in this study is available from the corresponding authors upon reasonable request." According to our Data Policy, data must either be included with the submission, be placed in a public repository, or available from a third party contact point who must not be an author on the manuscript. Please revise your data statement to a non-author institutional point of contact, such as a data access or ethics committee, and send this to us via return email. Please also include contact information for the third party organization (this contact should be a central address and not a named person), and please include the full citation of where the data can be found.

Thank you for bringing this to our attention. We updated the Data Availability statement as follows: “**The analyses carried out in this study included individual-level data, which cannot be made publicly available due to legal and ethical restrictions. To access the data, the approval must be obtained from the Scientific Advisory Committee of the Estonian Biobank and the Estonian Committee on Bioethics and Human Research. The inquiry for data should be sent via e-mail to** [**releases@ut.ee**](mailto:releases@ut.ee)**. For more details, please see the Data Access section at** [**https://genomics.ut.ee/en/content/estonian-biobank#dataaccess**](https://genomics.ut.ee/en/content/estonian-biobank#dataaccess)**. The source code used in this study is publicly available from GitHub (https://github.com/alterecho11/hc_use_estbb) and archived on Zenodo (https://doi.org/10.5281/zenodo.19615879).**” The statement names the non-author institutional contact points (the Scientific Advisory Committee of the Estonian Biobank and the Estonian Committee on Bioethics and Human Research), provides a central institutional e-mail address (releases@ut.ee), and includes a URL with full details on the data access procedure. No author is listed as a contact point. The code is publicly available on GitHub and permanently archived on Zenodo with a citable DOI. No access request or author contact is required for code.

2) We notice that your supplementary file “S1_Checklist.xlsx” is uploaded with the file type “other”. Please amend the file type to 'Supporting Information'.

We have now amended S1_Checklist as Supporting Information.

# Revision 1

# **We are most grateful for the time and care the Reviewers, Editor, and Editorial Team dedicated to reviewing our manuscript and for the thorough and considerate feedback. In response, we have revised the manuscript accordingly and believe we have substantially improved its clarity and precision. Our detailed responses to the reviewers’ and editorial comments are provided below.**

# Editorial Team

Please comment on potential selection bias by including only people covered by national health insurance.

In Estonia, approximately 95% of the total population has health insurance coverage through the Estonian Health Insurance Fund (EHIF) [36]. In the Estonian Biobank, approximately 99.5% of the participants have it [24].

We have now clarified in the Methods: “The cohort represents approximately 20% of the total adult population of Estonia (n_total_adult_ = 1,077,308 in 2025) [35]. Nearly all participants (99.5%) are insured by the Estonian Health Insurance Fund [24], which covers 95% of the total Estonian population [36], **suggesting the potential for selection bias due to insurance status is minimal**.”.

Moreover, we ask that you discuss as a limitation the lack of information regarding the underlying reasons for switching HCs, e.g. are there differences in cost even under insurance?

We thank the Editorial Team for this important remark. While differences in out-of-pocket expenses could not be captured, cost is unlikely to be a primary driver of switching or discontinuation. In Estonia, all hormonal contraceptives (HCs) are at least 50% reimbursed by the EHIF, with full package prices ranging approximately 5–34 EUR for short-acting HCs (pills, patch, ring) in 2026 [76]. For intrauterine devices (IUDs), while the upfront cost is higher (approximately 63–126 EUR), this represents a one-time expense rather than a recurring factor that would drive formulation switching. Based on personal communication with a gynaecologist, cost is rarely raised as a motivating reason during consultations.

We have now clarified in the Discussion: “**Additionally, though out-of-pocket expenses could not be captured and may have influenced usage trends and switching, this is unlikely to be substantial, given that EHIF reimburses at least 50% of the full price for HCs [76].**”.

Finally, can you please discuss if, in this 20 year period assesses, there were major changes in HC options that may drive such HC changes?

Detailed changes in the Estonian landscape of available HCs over the past 20+ years, such as shifts towards low-dose estrogen formulations, long-acting methods, and updated prescribing guidelines following EMA recommendations, have been comprehensively described by Kurvits et al [13].

To avoid redundancy with their work, but acknowledge the point, we have now added the following clarification to the Discussion: “**We note that the annual prevalence rates may reflect regulatory changes, prescription practices and market availability, as not all HC formulations were available throughout the whole study observation period. Specifically, over the 19-year study period, HC options in Estonia evolved in line with the EMA guidelines [10] and under regulatory oversight of the State Agency of Medicines (Ravimiamet) [68]. Key changes included a shift towards low-dose estrogen COCs, and improving access to long-acting methods and emergency contraceptives. Notably, subdermal implants got marketing authorisation in Estonia in December 2013, thereby diversifying the available contraceptive methods from 2014. Furthermore, shifts in clinicians’ thinking over the study period, such as broader use of IUDs in nulliparous women and POPs beyond lactating women, likely contributed to the observed trends.**”.

# Reviewer #1

Thank you for the opportunity to review the manuscript titled, "Hormonal contraceptive use trends in the Estonian Biobank: user profiles and a resource for genetic studies". My specific comments are below --

Page 2, lines 22-23: This may be a difference between US prescriptions and Estonian, but can the authors define "purchase data". Does this mean prescribing data? More detail would be helpful. Purchase data to me would mean over-the-counter or medications that can be purchases without a prescription from a physician.

Yes, in our dataset, all purchased data were prescribed data. In Estonia, all HCs except for emergency contraceptives require a prescription. However, focusing only on prescriptions would include medications that were prescribed but never purchased, potentially overestimating actual HC usage.

We have clarified in the title “Descriptive analysis of **prescribed hormonal contraceptive** use trends in the Estonian Biobank: user profiles and a resource for genetic studies” and in the text: “**In Estonia, all HCs except for emergency contraceptives require a prescription.**” and “This was a longitudinal descriptive study combining **prescribed** HC purchase data…”.

Page 2, line 30-31: Please provide the descriptive statistics for this sentence. Do you mean combined HCs dominated the total prescriptions? More information here would be helpful.

We thank the reviewer for bringing this to our attention. We agree that “combined HCs dominated” is not precise. We have now clarified: “Comparable to Estonian population, combined HCs dominated **usage among users aged 15-29**, while progestin-only HC usage increased with age and time.” The original sentence was based on the visual interpretation of Figure 1 (now Fig 2) and aimed to convey the overall trends briefly. Providing specific percentages for this sentence is challenging, given the age-stratified analysis, many years, and the ageing cohort. We hope this change addresses the reviewer’s concern while remaining concise in the Abstract.

Page 2, line 32: Provide the number of "users" for these percentages. It may also be good to differentiate between "users" and "participants" or use one term consistently.

Thank you for this comment, we have now added the numbers and used term “users” consistently: “Overall, 64.2% **(n = 46,920)** of users switched formulations at least once, with 17.7% **(n = 12,929)** being rapid switchers. Side effect-related diagnoses were recorded in 23.1% **(n = 2,982)** of rapid switchers, with excessive/irregular menstrual bleeding being most common. Genetic analysis revealed 5.3% **(n = 3,886)** of users carried at least one thromboembolism risk-increasing variant (3.5% **(n = 2,556)** carried Factor V Leiden only, 1.8% **(n = 1,276)** prothrombin G20210A only, and 0.07% **(n = 54)** both).”

Page 4, line 78-80: Please cite the WHO guidelines in this sentence.

We thank the reviewer for bringing this to our attention. We have now cited the WHO Medical Eligibility Criteria for Contraceptive Use (6th edition, 2025) [11] to support the statement.

Page 4, line 85: It would be helpful to provide the total number of participants in the Biobank - I assume this is not exclusively women?

Yes, the Estonian Biobank has both female and male participants. We have now clarified: “The Estonian Biobank (EstBB) is a volunteer-based population biobank with **212,000** participants **(~140,000 females,** 43% currently aged under 50**)**,…”.

Page 4, line 95: In all biobanks or specifically the EstBB?

Healthy participant bias has been observed in several widely used volunteer-based biobanks, such as UK Biobank, Estonian Biobank and Dutch Lifelines Study [ref]. We have now clarified: “However, previous studies have shown a “healthy participant bias” in **the widely used volunteer-based** biobanks…”.

Ref: Prictor M, Teare HJA, Kaye J. Equitable participation in biobanks: the risks and benefits of a "dynamic consent" approach. Front Public Health. 2018;6:253. pmid:30234093

Page 5, line 110: Provide the current total population of Estonia.

We have now added the total number of individuals aged 20 and above living in Estonia as of January 1, 2025: “The cohort represents **approximately** 20% of the total adult population of Estonia **(n_total_adult_ = 1,077,308 in 2025)** [35].”. The Statistics Estonia database provides information only for specific age groups (e.g., 10-14, 15-19, 20-24,…), so the presented number is a slightly conservative approximation of the total adult population.

Page 6, line 127L Just to confirm, the purchase invoice is the individual picking up the prescription at a pharmacy? Please clarify.

Yes, the purchase invoice records when an individual buys a package of prescribed HC from a pharmacy. We have now clarified in the Methods: “**The purchase invoice records the purchase of prescribed HC in a pharmacy**.”

Page 8, lines 168-175: Where these categories created by the author team or an already defined set of categories from other research?

We thank the reviewer for the opportunity to clarify. The terminology for rapid switchers and discontinuers was adopted from existing research on hormonal contraceptive use [43,44]. We defined non-switchers as a comparison/control group for future genetic analyses. The specific thresholds for all three groups (90 days for rapid switchers/discontinuers and 180 days for non-switchers) were based on the clinical guidelines that recommend an initial three-month “trial” period for HCs and the Estonian prescription system. Consequently, rapid discontinuers are individuals who used only one HC formulation and discontinued it within 90 days. Rapid switchers are those who used one HC formulation for up to 90 days and switched to another formulation within the next 90 days. Non-switchers are those who used only one HC formulation and had at least one usage period longer than 180 days. The 180-day threshold for non-switchers reflects both sufficient exposure and comparability with rapid switchers (who, by definition, have up to 180 days of observable HC-related activity), as well as the maximum prescription supply per one clinical visit in Estonia. Users exceeding 6-month threshold had to re-engage with the health system to continue HC use. These thresholds were applied consistently across all HC types to ensure comparability. In the current descriptive context, this approach produces clean, well-defined groups. For any hypothesis-testing study, sensitivity analyses varying the thresholds would be required or even using prospective design with a defined index date and follow-ups.

We have now clarified in the manuscript: “**While the terminology was adopted from existing research [43,44,ref], the thresholds were defined based on the clinical guidelines recommending an initial three-month ‘trial’ period and the Estonian prescription system.”**.

Ref: Ali MM, Cleland J. Contraceptive discontinuation, switching, abandonment and their reproductive consequences: an analysis of 1,539,071 episodes of reversible method use contributed from 61 countries that participated in DHS: population base-analysis. PLOS Glob Public Health. 2025;5(10):e0005174. pmid:41171726

Page 13: Thank you for the beautiful figures - I love them!

Thank you for this comment, it makes us so happy to read it!

Page 17, lines 322-341: This section is a mix of result findings and discussion. Please consider moving some of the interpretation of the data to the discussion.

We thank the reviewer for this suggestion. We have now updated S5 Appendix figure legend (“… **The number 13 is coloured red to mark the date when the Estonian government announced a state of emergency in 2020.**”) and the text as:

**Results**: “Furthermore, the overall monthly purchase trends differed notably for 2020 (Fig A in S5 Appendix), where March 2020 was 20% above the grand mean and April 2020 was 20% below it. Specifically, March had higher purchase numbers of CHC pills, patches, rings, and POPs than March 2019, with the peak on March 13th (Fig D in S5 Appendix). Conversely, LNG-IUDs and implants had their lowest record numbers in March and April 2020. Following June 2020, only 2nd generation COCs and LNG-IUDs had a slight increase in purchases compared to the corresponding periods in 2019. Also, compared to 2021, only 2nd generation and estradiol COCs, implants, LNG-IUDs and POPs returned to similar numbers as recorded in the pre-COVID-19 period.”

**Discussion**: “While we focused on purchasing and not prescription dates, we observed a higher number of CHC and POP purchases right before lockdown, **likely due to the stockpiling behaviour**, and a lower number during the first year of the COVID-19 pandemic, especially of implants and LNG-IUDs, **likely due to limited access to healthcare services**.”

Page 26, line 503: Self-report would be a disadvantage when it comes to specific symptom side effects of HC exposure.

In the specific sentence on page 26, line 503 (now page 31, lines 584-587), we were referring to self-report bias in reporting duration of use, age at first/last HC use, and similar HC-related covariates commonly used in research studies. We have now clarified “Additionally, **our data and approach for defining HC exposure using purchase records avoid** self-report and recall bias **in HC usage duration and timing**, which have been shown to increase errors and reduce research reproducibility significantly.”.

# Reviewer #2

The manuscript presents an interesting and comprehensive analysis of hormonal contraceptive (HC) use among participants from the Estonian Biobank. The study is well-written and represents a significant contribution to understanding real-world patterns of HC use. Notable strengths include the longitudinal approach, with an 18-year observation period, and the integration of application data with biobank records. In addition, the manuscript has a high relevance for clinical practice and public health.

Critical Comments and Suggestions for Improvement:

1. General Use Trend:

The authors note that not all HC formulations were available throughout the entire observation period (line 280ff.). It would be beneficial to clarify whether, and to what extent, this fact influences the annual prevalence rates.

We thank the reviewer for this suggestion. We acknowledge that the annual prevalence rates are partially influenced by market availability. As our dataset does not have information about specific HC brands, we are unable to directly link marketing authorisation dates to specific formulations, and therefore cannot quantify this effect precisely.

We have now noted in the Discussion: “**We note that the annual prevalence rates may reflect regulatory changes, prescription practices and market availability, as not all HC formulations were available throughout the whole study observation period.**”.

2. Contraceptive Switching:

The distinction between "rapid switchers" and "rapid discontinuers" is clear. However, Figure 3a shows that individuals switched between one and more than eight formulations. It would strengthen the manuscript if the authors analyzed potential side effects stratified by the number of switches.

Figure 3a (now Fig 4A) captures HC formulation switching across the entire observation period (broad switchers), where switching may occur for many reasons beyond experiencing side effects, and the time window between switches may vary considerably across individuals. Instead, we focus on rapid switchers, a more defined group in which side effect-driven behaviour is more plausible, and examine their potential side effect profiles using diagnoses recorded around the switching events. We find the reviewer’s suggestion genuinely intriguing and believe that a stratified analysis of this kind would be most informative if detailed HC side effect reports could be linked to HC usage periods.

We have mentioned similar in the Discussion: “…, we could not definitively determine specific reasons for switching between formulations or discontinuation. Future implementations of free-text data mining of unstructured EHRs could partially aid in mitigating this limitation.” and Conclusions: “Expanding on our approach, future studies could **analyse side effects stratified by the number of switches**, and investigate the impact of genetic factors on switching behaviour and HC-related side effects”.

3. Terminology:

The term "broad switcher" is used in the results and discussion sections but is not defined in the methods. Please clarify whether this term is synonymous with "switcher" or if it has a distinct meaning.

Thank you for bringing this to our attention. “Broad switcher” is an individual who used two or more different HC formulations over the whole study observation period.

We have now clarified this in the Methods: “Finally, all HC users were classified as **broad** switchers, rapid switchers, non-switchers and rapid discontinuers following these criteria: (a) **broad** switchers were individuals who used two or more different HC formulations during the study observation period, (b) rapid switchers…”.

4. Visualization of Switching Pathways:

Figure 3b, which illustrates switching pathways, is difficult to interpret due to the numerous changes and color coding. I recommend including the detailed information on HC switching behavior from Additional File 1 in the main text.

We have now moved text from the Additional File to the main text to aid the interpretation of the Figure 3b (now Fig 4B).

5. Side Effects and ICD-10 Diagnoses:

The manuscript should clarify whether the listed ICD-10 diagnoses appeared in the patient's medical records for the first time before the change in medication, or if they had already occurred before the first contraceptive was taken. Additionally, please specify what period prior to contraceptive use was considered.

We thank the reviewer for the opportunity to clarify. Yes, we always used first-time-ever records of ICD-10 diagnoses for potential side effects.

We clarified in the Methods (section Longitudinal diagnosis history and thromboembolism risk factors): “Second, when evaluating diagnoses during the HC usage period and up to three months after the usage period end date to identify diagnoses related to potential side effects and possible reasons for HC discontinuation, we **only considered first-time-ever diagnoses** to avoid misinterpretation from pre-existing conditions. **The same approach was used for diagnoses during the gaps** between two different HC usage periods of rapid switchers.”.

The period prior to contraceptive use was considered only for the first-time-observed HC usage period to identify potential non-contraceptive reasons for starting, and its duration was three months/90 days (“First, the records of clinical diagnoses in the **three-month window preceding the first-time-observed HC usage period** start date were used to identify potential non-contraceptive reasons for HC initiation…”).

Conclusion:

Overall, the manuscript is well-executed and provides valuable insights. Addressing the points above would further enhance the clarity and impact of the findings.

# Reviewer #3

This manuscript addresses an important public health topic and is based on a new and valuable nationwide dataset with substantial potential to inform contraceptive use patterns and safety. The manuscript is well written; therefore, my comments are intended to strengthen clarity of the manuscript.

The manuscript states that purchases with dosages <0.3 or >6 were excluded to avoid mistype errors. While the rationale is generally reasonable, the explanation should be more explicit linked to the Estonian dispensing and prescribing system. Specifically, it would be helpful to write if this rule applies uniformly across all HC products or depends on prescription duration, or clinical context.

We thank the reviewer for this important clarification. This rule applies only to short-acting hormonal contraceptives (HCs), as they are dispensed in standardised packages with predictable repeat purchasing. For long-acting reversible HCs, such as intrauterine devices (IUDs) and implants, this rule was not applicable, given their “single-purchase, single-use” nature. While isolated exceptions with larger HC stockpiling may exist, this is unlikely to affect the findings substantially.

We have now clarified in the Methods: “In the following filtering step, all entries of **short-acting HCs** where the purchased dosage was less than 0.3 or greater than 6 were excluded (n_purchases_ = 653 (0.04%)). **This had a negligible impact on prevalence estimates and analyses**, and ensured the dataset was free from mistype errors. **In the Estonian healthcare system,** buying less than a third of a package is not possible, and gynaecologists or **midwives** prescribe, per visit, a maximum of three prescriptions, each for two **short-acting HC** packages (i.e. for a six-month coverage period).”.

Although only 0.04% of purchases were excluded, it would be helpful to explicitly state that this filtering step had a negligible impact on prevalence estimates and downstream analyses, as even small exclusions can raise concerns about selection bias in population-based studies.

We have now clarified: “… **This had a negligible impact on prevalence estimates and analyses**, and ensured the dataset was free from mistype errors. **In the Estonian healthcare system,** buying less than a third of a package is not possible, and gynaecologists or **midwives** prescribe, per visit, a maximum of three prescriptions, each for two **short-acting HC** packages (i.e. for a six-month coverage period).”.

The adjustment of HC exposure periods using pregnancy-related ICD-10 codes require further information. For example, the authors should explain if HC exposure periods were truncated at the first pregnancy-related code, fully excluded, or handled in another way, and how multiple or overlapping pregnancy codes were treated.

Thank you for raising this important point. The inferred HC usage periods of all formulations were truncated at the earliest pregnancy-related ICD-10 diagnosis (O00–O84, O94–O99) falling within the inferred usage period. Postpartum complication codes (O85–O92) were excluded. When multiple pregnancy codes existed, the earliest date was used as the truncation point, and only one code was retained if several codes occurred on the same date. Backdating a fixed period before pregnancy diagnosis was not feasible, as pregnancy-related codes range from early losses (e.g., O03 at 6–12 weeks of gestation) to full-term deliveries (O80–O84 at ~40 weeks). The truncation at pregnancy diagnosis serves as a safeguard for cases where inferred coverage extended beyond it. In total, 3.4% (n = 8,541) of all usage periods were adjusted this way.

We have now clarified in the text: “**Inferred** HC usage periods were additionally censored with ICD-10 pregnancy codes (O00–O99, *excl.* O85–O92 (complications predominantly related to the puerperium)) that occurred within the original coverage period. **For example, if a pregnancy code appeared within an inferred HC usage period, we ended that period at the pregnancy diagnosis date. We did not backdate before the pregnancy date, as pregnancy-related diagnoses vary widely in gestational timing. This adjustment was applied across all HC types to avoid differential bias.**”

The statement that "usage periods were created using Node.js v18.12.1" is unclear and does not add meaningful methodological information. Therefore, the authors should explain what Node.js was used (e.g., execution of custom data-processing scripts, prescription linkage, exposure window construction), and if a bespoke script or algorithm was developed. If such a script exists, indicating if it is available (e.g., in supplementary materials or a repository), or explaining why it cannot be shared.

We developed a custom Node.js script to convert purchase data (HC formulation, dosage, package quantity, purchase date) into usage periods for computational efficiency. The script is explicitly designed to process Estonian Biobank data, with its specific data structures and identifiers. As medication data formats vary across countries and healthcare systems (e.g., differences in drug coding conventions, package sizes, dosing regimens, and the way dispensing records are recorded), the script would require substantial modifications to apply to other datasets. The methodological approach can be replicated in any programming language. We have now removed the sentence to avoid confusion.

The manuscript reports that BMI ranged from 14.1 to 43 with a mean of 24.3 during HC use. However, this range appears to reflect the predefined inclusion criteria rather than observed variability in the data. The wording should therefore indicate that this was the eligible BMI range.

We thank the reviewer for bringing this to our attention. In response to Reviewer 4, we have removed the demographic details from the text and added them to Table 1. According to your suggestion, we have noted in Table 1 footnote: “BMI range reflects predefined inclusion criteria (see Methods).”.

The authors should describe briefly in the text how BMI measurements were obtained in relation to HC use (e.g., closest measurement before first initiation or averaged over time) and if it was self-reported.

This has been mentioned in the Methods: “body mass index **(BMI) closest to the usage period start date**, were used for analysis. **BMI values originated from self-reported data or have been extracted from EHRs** and were categorised by Centers for Disease Control and Prevention criteria…”.

The authors observed that estimated HC prevalence rates were systematically higher than those reported by Kurvits, although the temporal trends were similar. To aid interpretation, the authors should explain briefly the key differences between the two studies in the text, such as age ranges, data sources, denominators, and definitions of HC use.

We thank the reviewer for this suggestion, as its addition substantially strengthens our work. We have now clarified in the Discussion: “Compared with Estonian national statistics (Kurvits *et al.*, 2021) [13], we had slightly higher prevalence rates for POCs and CHCs. **This could be due to the several methodological differences, such as differences in age range (15**–**49 vs 15**–**55 years), denominator (Estonian population vs Estonian Biobank female participants), and the definition of HC use, where we set maximum length for long-acting methods, included cyproterone/ethinylestradiol (CPA/EE) HC, and incorporated short pauses (<90 days between two purchases) as part of the usage period.** Also, EstBB 15–29 age groups have drastically decreased in size in recent years; therefore, the prevalence rates became “artificially” higher, as no new participants were recruited into EstBB after 2019. **Despite these differences, the similar temporal trends suggest that the overall pattern of HC usage is consistent.**”.

The authors reported a higher prevalence of thromboembolism risk factors among POC users. This finding reflects confounding by indication, as individuals with elevated thrombosis risk are preferentially prescribed progestogen-only methods. Without appropriate adjustment, comparisons between POC and CHC users are inherently biased. I suggest that the authors conduct additional analyses adjusting for age, BMI, smoking status, comorbidities and prior thrombosis when comparing the risk profiles of the HC groups.

Thank you for raising this important point and for the opportunity to clarify. We agree that confounding by indication contributes to the higher prevalence of many thromboembolism risk factors among POC users, as individuals with multiple risks are preferentially prescribed POCs. However, with our analysis, we did not intend to identify what drives prescribing decisions nor to establish causality between HC type and the incidence of thrombosis, as such questions from observational data require specific study designs, such as target trial emulation with careful adjustment for various covariates. Our aim was to descriptively characterise the risk profile of HC users across POC and CHC groups. Moreover, while confounding by indication explains why many risk factors are more prevalent among POC users, purchase of risk-increasing medications showed statistically higher prevalence among CHC users. This may indicate potential gaps in risk assessment during consultation, as well as areas for improvement.

We have now moved the sentence from Results to Discussion and elaborated further: “**Notably, thromboembolism risk factors were more prevalent among POC users than CHC users, reflecting confounding by indication, as individuals with multiple risk factors are preferentially prescribed POCs. However, the concomitant purchase of risk-increasing medications was statistically more prevalent among CHC users. This may indicate potential gaps in clinical risk assessment during prescribing consultations and highlight areas for improvement.**”.

The study would benefit from being stratified by estrogen dose. Given the well-established dose-dependent relationship between estrogen and thrombosis risk, stratifying by estrogen dose (e.g. ≤20 µg versus >20 µg ethinylestradiol, or other ranges) would enhance its clinical relevance.

We thank the reviewer for this constructive suggestion. We have now stratified the CHC user group by estrogen dose (low ethinylestradiol (low-ee), moderate ethinylestradiol (moderate-ee), and estradiol (e2) group) [34] and evaluated thromboembolism risk factors across these groups. Given the overall similar prevalence ratios and wider confidence intervals observed, especially for the smallest e2 CHC user group, presenting these stratified results in the main text would reduce the stability and clinical relevance of the effect estimates. We have therefore presented these results as Supplementary information (Table H in S3 Appendix and S6 Appendix), while retaining the overall POC and CHC comparison in the main text for more stable effect estimates.

**Results**: “The same analysis, stratified by estrogen dose (low ethinylestradiol, moderate ethinylestradiol, and estradiol CHC user groups), showed largely similar patterns across low and moderate ethinylestradiol groups (Table H in S3 Appendix and S6 Appendix). The estradiol group showed slightly higher prevalence ratios across several risk factors, though estimates should be interpreted cautiously given the small sample size and wide confidence intervals.”.

# Reviewer #4

This paper uses various existing databases to analyse the usage of hormonal contraception by women in Estonia.

I was asked for a methodological report and I interpret that to include all aspects of the design and conduct of the study.

**Points of detail**

Page 3 onwards Not really a statistical point but are so many references needed in the introduction? There are often three or four referring to the same detail.

We thank the reviewer for the comment. We have now selected the two most relevant studies where more than two citations supported the same detail.

Page 6 What percentage of the Estonian population is covered by the insurance fund?

According to the Estonian Health Insurance Fund, approximately 95% of the population is covered. Specifically, as of December 31, 2022, the insured population was 1,304,431 people (95.5% of the total population) [36]. In the Estonian Biobank, 99.5% of the participants is covered [24].

We have now clarified in the text: “The cohort represents approximately 20% of the total adult population of Estonia (n_total_adult_ = 1,077,308 in 2025) [35]. **Nearly all participants (99.5%) are insured by the Estonian Health Insurance Fund [24], which covers 95% of the total Estonian population [36], suggesting the potential for selection bias due to insurance status is minimal**.”.

Page 7 Did the age restriction only apply per purchase/prescription so that a woman could be in the cohort for other occasions when she was age–eligible?

Yes, the age restriction was applied only per purchase. We have now clarified in the text: “Finally, **purchases** for which the individual’s age at purchase was outside the 15–55 range (n_purchases_ = 1,306 (0.1%)) and individuals for whom BMI values closest to the HC usage start date were outside the range of 14–43 (n_users_ = 851 (1.1%)) were excluded.”.

Page 7 13.5, 19.5, 52 seems an odd progression especially when it is going to be reduced to 3, 5 and 6. Is there a typo here?

Numbers 13.5, 19.5, and 52 refer to milligrams of the progestin component (levonorgestrel) in intrauterine devices (IUDs). The dosage is not reduced to 3, 5, or 6, rather the usage period of the respective IUD can be up to 3, 5, or 6 years. For example, an IUD containing 13.5 mg of levonorgestrel can remain in place for up to 3 years.

Page 7 I am afraid I do not understand the sentence starting ‘HC usage period’. What does it mean for them to be adjusted? What is the force of the phrase ‘within the original coverage period’?

We thank the reviewer for highlighting this unclear phrasing. The verb “adjust” is not used here in a statistical sense. Our hormonal contraceptive (HC) usage periods are inferred from purchase data, and we use maximum coverage duration for each formulation. This approach can sometimes overestimate usage, especially for long-acting methods (e.g., IUDs have theoretical coverage 3–6 years depending on the dose), where removal is not reliably tracked. So, if a pregnancy code appears within an inferred HC usage period, we end that period at the pregnancy date. Backdating a fixed period before pregnancy diagnosis date was not feasible, as pregnancy-related codes range from early losses (e.g., O03 at ~6–12 weeks of gestation) to full-term deliveries (O80–O84 at ~40 weeks). Finally, to avoid introducing differential bias, we applied this adjustment uniformly to all HCs, not only long-acting ones.

We have now clarified in the text: “**Inferred** HC usage periods were additionally censored with ICD-10 pregnancy codes (O00–O99, *excl.* O85–O92 (complications predominantly related to the puerperium)) that occurred within the original coverage period. **For example, if a pregnancy code appeared within an inferred HC usage period, we ended that period at the pregnancy diagnosis date. We did not backdate before the pregnancy date, as pregnancy-related diagnoses vary widely in gestational timing. This adjustment was applied across all HC types to avoid differential bias.**”

Page 7 I do not understand the sentence starting ‘Furthermore, for HC’. This may reflect my general ignorance of hormonal contraception.

This is specific to HC pills. For example, some formulations have pills for 21 days followed by 7 placebo pills (or with 7 day pill-free interval), but the coverage period/the effect of the contraception is considered to be for 28 days. We simply acknowledged that we accounted for this in our HC usage period calculations.

Page 8 If I understand additional figure 1 correctly this would provide valuable information about how people are classified. However it tries to put much information onto one page with the result that it needs blowing up to read clearly. In turn that means you have to scroll repeatedly to get the information. I suggest that the section which has some similarity to a Gantt chart (towards the top) be made a separate figure and moved into the main text. The caption could also give more detail.

We thank the reviewer for this suggestion. We have now split Additional Figure 1 into two figures: the Gantt-chart-style flowchart is now Fig 1 in the main text with a detailed caption, and the remaining components are in S1 Appendix.

Page 11 It is clearly a good idea to credit the software used but just stating R base and the tidyverse is not that helpful. Base R contains many functions if we include the stats and graphics packages and the main tidyverse package downloads many functions which do the actual work. I would just cite the key packages used for analysis with proper citations which, as I am sure the authors are aware, can be found on CRAN or by using the citation() function.

We have now outlined all relevant R packages used for data analysis and visualisation in the manuscript [48–59]. We provide the detailed list of packages, versions, and citations here as well:

--- data.table | version: 1.15.4 ---

Barrett T, Dowle M, Srinivasan A, Gorecki J, Chirico M, Hocking T (2024). _data.table: Extension of `data.frame`_. R package version 1.15.4, <URL: https://CRAN.R-project.org/package=data.table>.

--- dplyr | version: 1.1.4 ---

Wickham H, François R, Henry L, Müller K, Vaughan D (2023). _dplyr: A Grammar of Data Manipulation_. R package version 1.1.4, <URL: https://CRAN.R-project.org/package=dplyr>.

--- tidyr | version: 1.2.0 ---

Wickham H, Girlich M (2022). _tidyr: Tidy Messy Data_. https://tidyr.tidyverse.org, https://github.com/tidyverse/tidyr.

--- stringr | version: 1.5.1 ---

Wickham H (2023). _stringr: Simple, Consistent Wrappers for Common String Operations_. R package version 1.5.1, <URL: https://CRAN.R-project.org/package=stringr>.

--- ggplot2 | version: 3.5.1 ---

Wickham H (2016). _ggplot2: Elegant Graphics for Data Analysis_. Springer-Verlag New York. ISBN 978-3-319-24277-4, <URL: https://ggplot2.tidyverse.org>.

--- lubridate | version: 1.9.3 ---

Grolemund G, Wickham H (2011). “Dates and Times Made Easy with lubridate.” _Journal of Statistical Software_, *40*(3), 1-25. <URL: https://www.jstatsoft.org/v40/i03/>.

--- formattable | version: 0.2.1 ---

Ren K, Russell K (2021). _formattable: Create 'Formattable' Data Structures_. R package version 0.2.1, <URL: https://CRAN.R-project.org/package=formattable>.

--- patchwork | version: 1.2.0 ---

Pedersen T (2024). _patchwork: The Composer of Plots_. R package version 1.2.0, <URL: https://CRAN.R-project.org/package=patchwork>.

--- ggalluvial | version: 0.12.5 ---

Brunson JC, Read QD (2023). “ggalluvial: Alluvial Plots in 'ggplot2'.” R package version 0.12.5, <URL: http://corybrunson.github.io/ggalluvial/>.

Brunson JC (2020). “ggalluvial: Layered Grammar for Alluvial Plots.” _Journal of Open Source Software_, *5*(49), 2017. doi: 10.21105/joss.02017 (URL: https://doi.org/10.21105/joss.02017).

--- viridis | version: 0.6.5 ---

Garnier, Simon, Ross, Noam, Rudis, Robert, Camargo, Pedro A, Sciaini, Marco, Scherer, Cédric (2024). _viridis(Lite) - Colorblind-Friendly Color Maps for R_. doi: 10.5281/zenodo.4679423 (URL: https://doi.org/10.5281/zenodo.4679423), viridis package version 0.6.5, <URL: https://sjmgarnier.github.io/viridis/>.

--- scales | version: 1.3.0 ---

Wickham H, Pedersen T, Seidel D (2023). _scales: Scale Functions for Visualization_. R package version 1.3.0, <URL: https://CRAN.R-project.org/package=scales>.

--- epitools | version: 0.5.10.1 ---

Aragon T (2020). _epitools: Epidemiology Tools_. R package version 0.5-10.1.

Page 11 It might be helpful to give some quantiles for variables like age. One thing which seems to be missing from the write–up is a table of demographic characteristics. Incidentally I do not think presenting tables in spreadsheets is appropriate for publication compared to properly typeset tables. By all means give us data in machine–readable format as well.

We thank the reviewer for these helpful suggestions. We have now added median and interquartile range (IQR) for age throught the manuscript, along with a table of demographic characteristic as a Table 1.

Regarding Supplementary table formatting, we have now reformatted all tables to improve readability and presentation quality. For those who wish to use data, tables are machine-readable in R using *readxl* package [ref]. For example, to read Table B from S3 Appendix:

install.packages("readxl")

library(readxl)

df <- read_excel("S3_Appendix.xlsx", sheet = "Table B", skip = 1),

where the skip argument accounts for the number of rows preceding the table header.

Ref: Wickham H, Bryan J. readxl: Read Excel Files. R package version 1.4.5. 2025. Available from: <https://CRAN.R-project.org/package=readxl> [accessed: 28/02/2026].

Page 16 What definition of outlier is being used here? If these are boxplot outliers then is that sensible with distributions which do not seem symmetrical (on the log scale)? The caption does not mention what I take to be the region between the upper and lower quartile.

Outliers are defined using the standard boxplot convention (values beyond 1.5×IQR from the Q1 and Q3 quartiles), as implemented by the ggplot2 geom_boxplot() function. We used a log scale because our research interest focuses on individuals with short usage periods, i.e., those who rapidly discontinue or switch HCs. Without a log scale, the visualisation would be dominated by the right tail of long-term users, obscuring the variation most relevant to our analysis. We acknowledge that this compresses the right tail and have noted the log scale explicitly on the axis.

We have now clarified in the Figure 2 (now Fig 3) caption: “**The box represents the interquartile range (25th to 75th percentile),** with the central diamond shapes marking the **median**, and the black dots marking the outliers.”.

Page 21 We really need estimates of differences with confidence intervals for all of these. The same applies on page 2. This applies especially to those which failed to reach a conventional level of statistical significance (Chan, 2013). Editors of health journals started calling for measures of effect size instead of p–values more than three decades ago (Langman, 1986; Gardner and Altman, 1986) For some reason sensitivity and specificity do, correctly, have confidence intervals.

We thank the reviewer for this comment. We have now added 95% confidence intervals (CI) throughout the manuscript. Specifically, for comparisons of thromboembolism risk factor prevalence between HC groups, for consistency, we reported prevalence ratios (PR) with 95% CIs, as these provide more interpretable effect estimates when outcomes are common (>10%). For other categorical associations, we reported odds ratios (ORs) with 95% CIs.

Page 21 Why use Fisher’s exact test for such large numbers?

We used Fisher’s exact test for all comparisons of categorical variables because it provides exact p-values and is computationally feasible. Some comparisons involved low expected counts, which can violate chi-square test assumptions, so we uniformly applied the exact test for consistency.

Page 27 I do not understand the apparent conflict between different medications for emergency prescription, over the counter versus on prescription only.

In Estonia, emergency contraceptives are hormonal contraceptives sold without prescriptions. Our dataset includes only purchases of prescribed HCs, therefore, emergency contraceptives are not captured in our study. We have simply stated this limitation in our manuscript.

We have now clarified in the text: “Emergency contraceptives are **sold** “over-the-counter” in Estonia, and our input was **purchases of prescribed HCs**.”.

The appropriate reporting guideline here would seem to be RECORD (Benchimol et al., 2015). I have not checked for compliance with all the individual items in the checklist.

We thank the reviewer for bringing this to our attention. We have now provided STROBE and RECORD checklists in the Supporting information S1 Checklist.

**Point of more substance**

This is a longitudinal dataset but there are features of the design which do not seem to have been taken into account. The percentages quoted throughout do not seem to have been adjusted for the fact that a woman may appear multiple times and so those observations are not independent. Also women may appear in the dataset in different age categories as they give observations and that needs attention too. If, in fact, the analysis did take that into account then the methods section needs to specify how it was done.

We thank you for raising this important methodological point. We have carefully examined our dataset, revised and updated the code, and updated the manuscript.

In general, the longitudinal structure of the dataset was used to identify active users in each calendar year. For describing changes in HC use trends over time, longitudinal samples with high year-to-year overlap are preferable to independent cross-sectional samples, as they allow more precise estimation of temporal changes by reducing between-sample variability. In our dataset, women contributing to consecutive annual estimates within the same age group are largely the same individuals, and this high overlap means that observed year-to-year changes in HC use reflect genuine shifts in prescribing patterns, women’s preferences, and market availability rather than sampling noise. This provides greater precision in describing short-term trends than would be achievable with independent random samples of equivalent size.

The updated percentages presented in the section titled “General use trends” reflect cross-sectional distributions of total covered days (i.e. active use) by HC type in each calendar year, stratified by age group. Each woman’s active years were derived by expanding her inferred HC usage periods across all calendar years falling between her start and end of coverage. Within each calendar year, an age group was assigned based on the simplified constructed age (active usage year minus the birth year), ensuring that each woman contributed to exactly one age group per calendar year. We verified this in the dataset, and no woman appeared in more than one age group within the same calendar year. Women using multiple HC types within the same calendar year contribute to each relevant HC type’s proportion, reflecting clinical reality. The proportions in Figure 1c (now Fig 2C), therefore, reflect the distribution of total covered days by HC type, rather than the percentage of unique users per HC type or percentage of unique woman-HC type pairs (as in the previous version of the manuscript). Next, in the descriptive section “Hormonal contraceptive switching”, the reported percentages reflect the unique number of switchers within the HC type divided by the total number of rapid switchers. In response to the above-mentioned, we have now revised the relevant Methods section, Results, and Figure labels and captions.

Finally, for the analysis of thromboembolism risk factors, conducted only for the year 2022, women using multiple HC types were assigned to the first observed HC type in 2022, ensuring one observation per woman. In regard to this comment and your previous comments (e.g. use of CIs), we have now revised the whole section “Presence of thromboembolism risk factors”.

While the conclusions of the study remain unchanged, your feedback helped us improve the clarity and precision of the work, for which we are most grateful.

**Summary**

Several points for clarification and a more important one about the relationship between the design and the analysis.

Michael Dewey

**References**

E I Benchimol, L Smeeth, A Guttmann, K Harron, D Moher, I Petersen, H Sørensen, E von Elm, S Langan, and RECORD working committee. The REporting of studies Conducted using Observational Routinely–collected health Data(RECORD) Statement. PLOS Medicine, 12(10), 2015.

L S Chan. Minimal clinically important difference MCID adding meaning to statistical inference. American Journal of Public Health, 103:e24–e25, 2013.

M J Gardner and D G Altman. Confidence intervals rather than P values: estimation rather than hypothesis testing. British Medical Journal, 292: 746–750, 1986.

M J S Langman. Towards estimation and confidence intervals. British Med- ical Journal, 292:716, 1986.

# Technical Requests

1) Please ensure that the funders and grant numbers match between the Financial Disclosure field and the Funding Information tab in your submission form. Note that the funders must be provided in the same order in both places as well.

- State the initials, alongside each funding source, of each author to receive each grant.

- State what role the funders took in the study. If the funders had no role in your study, please state: “The funders had no role in study design, data collection and analysis, decision to publish, or preparation of the manuscript.”

We have now added authors’ initials alongside each funding source and a statement that the funders had no role in the study. We confirm that the information is presented in the same order across all documents.

2) We ask that a manuscript source file is provided at Revision. Please upload your manuscript file as a .doc, .docx, .rtf or .tex.

We have now provided all text files in .docx format.

3) Please provide separate figure files in .tif or .eps format. For more information about figure files please see our guidelines:

<https://journals.plos.org/plosmedicine/s/figures>

We have verified all Figures using the suggested PLOS’s NAAS tool (<https://ngplosjournals.pagemajik.ai/artanalysis>) and provided them in .tif format.

4) We do not publish any copyright or trademark symbols that usually accompany proprietary names, eg ©, ®, ™ (e.g. next to drug or reagent names). Please remove all instances of trademark/copyright symbols throughout the text, including: ® on pages 7 and 34.

As response to Reviewer 3, we have already removed the sentence and the citation containing the symbol.
